# Emergence of Increased Resistance and Extensively Drug-Resistant Tuberculosis Despite Treatment Adherence, South Africa

**DOI:** 10.3201/eid1602.090968

**Published:** 2010-02

**Authors:** Alistair D. Calver, Alecia A. Falmer, Megan Murray, Odelia J. Strauss, Elizabeth M. Streicher, Madelene Hanekom, Thelma Liversage, Mothusi Masibi, Paul D. van Helden, Robin M. Warren, Thomas C. Victor

**Affiliations:** West Vaal Hospital, Orkney, South Africa (A.D. Calver, T. Liversage, M. Masibi); Stellenbosch University, Tygerberg, South Africa (A.A. Falmer, O.J. Strauss, E.M. Streicher, M. Hanekom, P.D. van Helden, R.M. Warren, T.C. Victor); Harvard School of Public Health, Boston, Massachusetts, USA (M. Murray); 1 These authors contributed equally to this article.

**Keywords:** *Mycobacterium tuberculosis*, extensively drug-resistant TB, XDR TB, MDR TB, transmission, molecular epidemiology, DOTS, tuberculosis and other mycobacteria, South Africa, research

## Abstract

We investigated the emergence and evolution of drug-resistant tuberculosis (TB) in an HIV co-infected population at a South African gold mine with a well-functioning TB control program. Of 128 patients with drug-resistant TB diagnosed during January 2003–November 2005, a total of 77 had multidrug-resistant (MDR) TB, 26 had pre–extensively drug-resistant TB (XDR TB), and 5 had XDR TB. Genotyping suggested ongoing transmission of drug-resistant TB, and contact tracing among case-patients in the largest cluster demonstrated multiple possible points of contact. Phylogenetic analysis demonstrated stepwise evolution of drug resistance, despite stringent treatment adherence. These findings suggested that existing TB control measures were inadequate to control the spread of drug-resistant TB in this HIV co-infected population. Diagnosis delay and inappropriate therapy facilitated disease transmission and drug-resistance. These data call for improved infection control measures, implementation of rapid diagnostics, enhanced active screening strategies, and pharmacokinetic studies to determine optimal dosages and treatment regimens.

## Abstract 2

Improved infection control, rapid diagnostic tools, enhanced screening strategies, and pharmacokinetic studies are needed.

The emergence of drug-resistant tuberculosis (TB) is often attributed to the failure to implement proper TB control programs and correctly manage TB cases (*1*,*2*). Consequently, >450,000 multidrug-resistant (MDR) TB cases (resistant to at least isoniazid and rifampin) are estimated to occur globally each year, of which 1%–2% occur in South Africa (*3*,*4*). A recent survey conducted by the World Health Organization (WHO) and the Centers for Disease Control and Prevention (Atlanta, Georgia, USA) estimated that 7% of MDR TB samples were also extensively drug-resistant (XDR) TB (*5*), i.e., resistant to isoniazid and rifampin and to at least 1 representative of each class of the most effective second-line drugs (i.e., fluoroquinolones and the injectable drugs kanamycin, amikacin, or capreomycin). Concern over XDR TB was further heightened with the identification in 2006 of an XDR TB outbreak involving 53 cases in South Africa (*6*). This outbreak had an exceptionally high proportion of deaths among HIV co-infected case-patients and demonstrated the need for improved basic TB control measures (*7*) and enhanced infection control. Subsequently, those involved in investigating the outbreak suggested that, in the absence of drug-susceptibility testing (DST), the evolution of MDR TB and XDR TB was inevitable (*8*). A recent study in Uzbekistan showed the emergence of XDR TB while patients were being treated for MDR TB, which suggests that treatment regimens should be optimized and strategies developed for administering these regimens safely and effectively (*9*).

In 1999, WHO put forward the directly observed treatment short course (DOTS)–plus strategy, which proposed that the diagnosis and treatment of MDR TB could complement a well-functioning DOTS program and thereby control the emergence and spread of TB (*10*). These strategies have been implemented since 2000 by the health service at a gold mine in the North West Province in South Africa. Furthermore, a policy of biannual chest radiographic screening has been instituted, which contributes to the early identification of patients with active pulmonary TB (PTB). Using these rigorous case-finding and treatment strategies, the program has been able to achieve successful treatment outcomes in >85% of new sputum smear–positive TB cases since 2001 and an average of 77.2% successful for retreatment of smear-positive cases (A.D. Calver, unpub. data). Despite this success, the incidence of drug-susceptible TB has continued to rise, an increase that reflects both the rising HIV prevalence in this community and the occupational risks specific to the mine setting such as silicosis, congregate living, and working conditions. In 2003, a marked increase (2.4×) in the number of case-patients with DR TB was noted at this gold mine. We investigated this outbreak using a molecular epidemiologic approach and clinical and epidemiologic data to identify inadequacies in the implemented DOTS-plus strategy that lead to the emergence of pre–XDR TB (MDR TB with resistance to either kanamycin or ofloxacin [*11*]) and XDR TB.

## Materials and Methods

### Study Population

This study was conducted at a gold mine in South Africa, January 2003–November 2005. Any employee with a new lesion detected on biannual occupational health chest radiographic screening is referred to the hospital. Similarly, all persons with self-reported suspected TB and unexplained weight loss, unexplained persistent cough for >2 weeks, and unexplained night sweats are also referred for TB investigation. A bacteriologic diagnosis of PTB was made by auramine-O fluorescent stain microscopy of 4 concentrated sputum smears and 2 TB cultures using Mycobacterial Growth Indicator Tube (MGIT) (BD Diagnostic Systems, Franklin Lakes, NJ, USA). Patients who had negative smear and culture results and chest radiographic results suggestive of PTB were monitored with repeat sputum smears. They were treated for TB only if other causes for the lesion could not be found and the patients’ symptoms and radiographic results deteriorated. DST for isoniazid and rifampin was performed on all positive *Mycobacterium tuberculosis* cultures by using MGIT. Time from seeking treatment until diagnosis of drug resistance (isoniazid and rifampin) ranged from 21 to 112 days. Second-line DST was done for ethambutol, ofloxacin, and kanamycin in MGIT 960 media containing 5 µg/mL, 2 µg/mL, and 4 µg/mL, respectively. Pyrazinamide DST was carried out according to the BACTEC manual (BD Diagnostic Systems) (*12*).

Patients with bacteriologically confirmed cases of TB were treated according to WHO/International Union Against Tuberculosis and Lung Disease guidelines. They were treated within the hospital to limit community transmission until sputum smears were negative for 2 consecutive specimens collected on separate days (checked weekly). Thereafter, patients received supervised outpatient treatment, and adherence was monitored by observing the patient receive and swallow the issued daily doses. Adherence rates were reported to the mine healthcare service management and ranged from 95% to 98%.

Patients with a diagnosis of MDR TB were hospitalized and treated based on current DOTS-plus guidelines from the South African National TB control program. Treatment regimens included at least 4 drugs and were based previous treatment history and DST. Injectable drugs were stopped when sputum cultures were negative for at least 2 successive months or when side effects necessitated discontinuance. Once sputum cultures had been negative for at least 3 successive months, patients were discharged to outpatient treatment. Oral medication was continued for at least 12 months after the first negative culture, with a minimum total duration of 18 months. An outcome of cure was assigned to patients with MDR TB or XDR TB if they maintained culture conversion and completed a full course of treatment for >18 monthsTransferred out was defined as a patient who was transferred to another healthcare facility for further TB treatment.

### Infection Control

Patients with positive smear cultures were admitted to a TB ward, which is equipped with UV lights and is separated from the rest of the hospital wards by a 100-m cross-ventilated corridor. The windows in the TB ward are open throughout the year, creating good natural cross-ventilation. Although patients were advised to avoid close contact with patients from other wards, patients were not confined to this ward, and some contact may have occurred within the hospital grounds. In addition, some patients were diagnosed with PTB while they were hospitalized and were being assessed for other conditions. Such patients were subsequently transferred to the TB ward. Before 2004, patients with DR TB were hospitalized in a miniward within the TB ward used for patients with drug-susceptible disease. However, in response to the increase in the number of MDR TB patients, a separate MDR TB ward was opened in 2004. This ward is separated from the rest of the hospital by electronically locked doors for restricted entry and exit, is fitted with ceiling mounted UV air sterilizing units, and patients leaving the ward are fitted with a PF95 mask. However, patients are kept on the general TB ward until drug-resistant disease is confirmed, at which point they are transferred to the MDR TB ward.

### Participant Enrollment

All mine employees and dependents with drug-resistant TB diagnosed during January 2003–November 2005 were included in this study (average number of persons covered per year: 28,943 in 2003, 25,541 in 2004, and 21,790 in 2005). Clinical and demographic data were collected retrospectively and included the following: age, sex, site of disease (PTB or extrapulmonary TB), sputum smear results, treatment category (new or retreatment cases), TB outcome, HIV status (despite extensive patient education and counseling, there are reluctance and denial issues relating to HIV testing), antiretroviral treatment (ART), place of residence within the mine area, and dates and locations of hospital stays. This study was approved by the ethics committee (internal review board) of Stellenbosch University, Tygerberg, South Africa.

### Genotyping

Drug-resistant *M. tuberculosis* isolates were genotyped by insertion sequence (IS) *6110* restriction fragment-length polymorphism (RFLP) (*13*), spoligotyping (*14*), and mycobacterial interspersed repetitive unit (MIRU) typing (12-loci format) (*15*). The *katG*, *rpoB*, *pncA*, *embB,* and *gyrA* genes of the *M. tuberculosis* isolates were sequenced by using the ABI PRISM DNA sequencer (Applied Biosystems, Foster City, CA, USA) to identify nonsynonymous single nucleotide polymorphisms conferring isoniazid, rifampin, pyrazinamide, ethambutol, and ofloxacin resistance, respectively (*16*). Strains that share an identical genotype (spoligotype, IS*6110* RFLP, and MIRU type for low copy-number strains) were classified as clustered; clustered strains were considered to be part of an ongoing chain of transmission. Isolates with unique strain genotypes from new cases-patients with drug-resistant TB were considered to have primary resistance, whereas isolates with unique strain genotypes from patients undergoing retreatment were thought to have either acquired resistance during therapy or to be a reactivated a drug-resistant strain (*17*).

To elucidate the molecular evolution of drug resistance within a transmission chain, we conducted a phylogenetic analysis using DNA sequence data from isolates from the single large cluster detected through genotyping. We used 2 distinct algorithms: 1) the heuristic parsimony algorithm, and 2) the neighbor-joining distance algorithm in conjunction with sampling the original dataset with replacement to construct a series of 1,000 bootstrap replicates of the same size as the original dataset (PAUP 4.0* software version 4; Sinauer Associates, Sunderland, MA, USA) (*18*). A consensus tree was generated by using the program CONTREE (PAUP 4.0*) in combination with the majority rule formula.

### Contact Tracing

Hospital and employment records were reviewed to identify potential sites of contact between patients in the largest cluster. We considered patients to have been exposed to MDR TB within the hospital setting if they been previously admitted to the hospital before their admission for diagnosis with MDR TB, during which time a patient with active MDR TB of an identical genotype had also bee also hospitalized. Patients were considered to have had work contact if they had worked the same mine shaft as a person with an MDR TB diagnosis, and to have had residential contact if they lived in the same building or group of buildings before MDR TB diagnosis.

### Statistical Analysis

For univariate analyses of clustering, we used logistic regression to estimate odds ratios (ORs) and 95% confidence intervals (CIs). We calculated p values by using the Mantel-Haenszel χ^2^ method or Fisher exact test. Statistical tests were 2-sided. We used a multivariate logistic regression model to control for possible confounders. We also used a logistic regression model to estimate the OR of death among patients infected with clustered strains. Analyses were performed with STATA software version 9.0 (StataCorp LP, College Station, TX, USA) and SAS version 9.1 (SAS Institute, Cary, NC, USA).

## Results

During the study period, 3,003 patients with TB were notified; 1,443 (48%) had new PTB cases, 755 (25%) were being re-treated for PTB, and 805 (27%) patients had extrapulmonary TB. Of these case-patients, 70% sought treatment on their own at healthcare clinics or hospital with symptoms, while the remaining 30% were identified by active screening. Successful treatment (cure or treatment completed) was achieved in 86.5% of all TB case-patients during the study. Less than 2% of TB case-patients defaulted or had an unsuccessful treatment outcome, and 12% of TB case-patients died as a result of TB or other causes. One hundred twenty-eight (4.3%) TB case-patients had drug-resistant TB; of those, 13 (10.2%) were diagnosed with isoniazid-resistant TB, 7 (5.6%) with poly–drug-resistant TB, and 108 (84.4%) with MDR TB. Among isolates identified as MDR TB, 26 were pre–XDR TB and 5 were XDR TB.

Table 1 summarizes demographic and clinical characteristics of the cohort of 128 patients with drug-resistant TB. All of the employees had worked at the mine for at least 6 months (median 15.0 years, range 0.5–27 years) and had passed a preemployment physical examination that ruled out active TB. Among those who were HIV seropositive, 60 (70.4%) had smear-positive TB and 52 (62.0%) had a CD4 count of <200 cells/mm^3^ (median 74 cells/mm^3^). Fifty-six (66.6%) of those seropositive for HIV were receiving HIV education from the wellness HIV clinic; 7 (8.3%) had initiated ART before being diagnosed with drug-resistant TB, and 22 (26.2%) had initiated ART after being diagnosed with MDR TB. Among those who started ART after being diagnosed with drug-resistant TB, the median time from diagnosis to initiation of ART was 172 days (range 41–1,425 days).

**Table 1:** Characteristics of mine workers with drug-resistant TB diagnosed January 2003–January 2005, South Africa*

Characteristic	No. (%) HIV+ workers	No. (%) HIV– workers	No. (%) workers with unknown HIV status	Total no. (%) workers
Total	84	7	37	128
Age (average)	43	43	42	43
Sex				
M	82 (97.6)	7 (100)	35 (94.6)	124 (96.9)
F	2 (2.4)	0	2 (5.4)	4 (3.1)
Case definition				
New	38 (45.2)	0	18 (48.6)	56 (43.7)
Retreatment†	46 (54.8)	7 (100)	19 (51.4)	72 (56.3)
Sputum smear‡				
Positive	60 (70.4)	4 (57.1)	31 (83.8)	95 (74.2)
Negative	24 (28.6)	3 (42.9)	5 (13.5)	32 (25)

Drug-resistance phenotype				
Isoniazid (mono) resistant	8 (9.5)	1 (14.3)	4 (10.8)	13 (10.1)
Poly resistant	4 (4.8)	0	3 (8.1)	7 (5.5)
MDR	72 (85.7)	6 (85.7)	30 (81.1)	108 (84.4)
Pre–XDR	22 (26.2)	3 (42.9)	1 (2.7)	26 (20.3)
XDR	2 (2.4)	1 (14.3)	2 (5.4)	5 (3.9)
Outcome				
Treatment completed or bacteriologic cure	26 (30.1)	3 (42.9)	11 (29.7)	40 (31.3)
Failed	1 (1.3)	0	0	1 (0.8)
Died	31(36.9)	2 (28.6)	12 (32.4)	45 (35.2)
Transferred out	20 (23.8)	2 (28.6)	10 (27.0))	32 (25.0)
Lost to treatment§	6 (7.1)	0	4 (10.8)	10 (7.8)
CD4 cell count, cells/mm^3^¶				
<200	52 (62)	ND	5 (13.5)	57 (44.5)
>200	18 (21.4)	2 (28.6)	ND	20 (15.6)

*TB, tuberculosis; MDR, multidrug-resistant; XDR, extensively drug-resistant; ND, not determined. †All prior TB episodes were treated at the mine. ‡1 sputum smear result missing. §One had monoresistant TB and 9 had MDR TB. ¶CD4 counts available for 77 patients only.

Outcomes were generally poor, with only 31.3% completing treatment with confirmed bacteriologic cure. Forty-five (35.2%) patients died; 7 (15.6%) of those who died did not have a confirmed TB diagnosis at time of death, 12 (26.7%) had been diagnosed with TB but were receiving standard therapy at the time of death, and 26 (57.8%) case-patients who were receiving MDR TB treatment died. Of note, >50% of the deaths were due to other AIDS-related conditions. Among those who died, median time to death from beginning of treatment was 5 months (range 1–24 months).

Combined genotype analysis of isolates from 124 of the 128 case-patients identified 61 distinct drug-resistant genotypes (Appendix Figure). Fifty isolates were unique and 74 were clustered. Among the 11 clusters, the cluster size ranged from 2 to 42. We estimate that at least 63 (85.1%) of the 74 clustered isolates had primary drug resistance, assuming that each cluster was initiated by an isolate that acquired drug resistance (*19*). Among the 50 unique genotypes, 25 (50%) were cultured from new case-patients, which suggests primary drug resistance. Accordingly, we suggest that 71% of drug-resistant TB cases resulted from transmission of preexisting drug resistant strain.

Clustering was more frequent among case-patients with MDR TB than among those with monoresistant or polyresistant strains (unadjusted OR 14.20, p = 0.001; adjusted OR 14.13, p = 0.002) (Table 2). When we compared clustering among pre–XDR TB and XDR TB isolates and those with monoresistance or polyresistance, we found that these highly resistant strains were also more likely to be in a cluster than those with less resistance (unadjusted OR 27.42, p<0.001). Twenty (76.9%) of the pre–XDR TB strains and 4 (80%) of the XDR TB strains clustered with circulating MDR TB strains. Table 2 illustrates that additional risk factors for clustering were not identified in either the univariate or multivariate analysis, although patients in clusters were more likely to die than those whose isolates were not clustered (unadjusted OR 2.28, p = 0.04; adjusted OR 4.76, p = 0.007). Of note, 59% of clustered case-patients had a documented previous episode of TB, which suggests reinfection with a circulating strain (Table 2). Although higher CD4 counts were associated with less clustering (unadjusted OR 0.49, p = 0.19; adjusted OR 0.51, p = 0.28), this association did not reach significance.

**Table 2:** Patient risk factors for having clustered TB isolates, January 2003–January 2005, South Africa*†

Category	Cluster status		Univariate OR	p value	Multivariate OR	p value
	Unique, n = 50	Clustered, n = 74				
Treatment history						
Re-treatment	25	44				
New case	25	30	1.47	0.29	0.69	0.50
Sex						
M	49	71				
F	1	3	1.27	0.52	NI	
Age, y						
<45	33	51				
>45	17	23	0.87	0.73	NI	
HIV status						
Negative	4	3				
Positive	29	53	2.43	0.26	2.33	0.33
Sputum smear						
Negative	14	19				
Positive	36	55	1.12	0.77	NC	
MDR TB						
Mono or poly resistant	16	2				
INH and RIF resistant	34	72	14.2	0.001	14.13	0.002
MDR plus						
Mono or poly resistant	16	2				
Pre–XDR TB/XDR TB	7	24	27.42	<0.001	NC	
Died						
No	38	42				
Yes	12	32	2.28	0.04	4.76‡	0.007‡
CD4 count, cells/mm^3^						
<200	15	42				
>200	8	11	0.49	0.19	0.51§	0.28

*TB, tuberculosis; OR, odds ratio; CI, confidence interval; NI, variables not included in final logistic regression model based on model selection criteria; NC, variables a priori not considered in logistic regression model; MDR, multidrug-resistant; XDR, extensively drug-resistant; INH, isoniazid; RIF, rifampin. †Logistic regression performed on 71 observations for which HIV and CD4 available, model adjusted for treatment history, HIV, CD4, and MDR. ‡Logistic regression on probability of death given clustering, adjusting for HIV, MDR-plus, and age. §CD4 counts available for 77 patients only.

**Figure 1:**
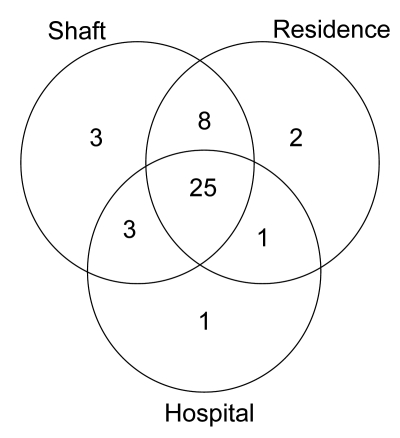
Venn diagram of number of potential contacts by type among patients in the largest multidrug-resistant tuberculosis (MDR TB) cluster, South Africa, 2003–2005. Each circle represents potential places of contact: shaft, mine shaft (work); residence, place of residence; hospital, hospitalization at the same time as another MDR TB case-patient.

Figure 1 demonstrates that most patients in the largest cluster had multiple different types of contact; 32 (76.2%) had a non–MDR TB hospitalization at the same time another patient in the cluster was admitted for MDR TB. Thirty-nine (92.9%) patients worked in a shaft in which another MDR TB patient in the cluster had worked, and 36 (85.7%) of the patients resided in the same residential unit where another MDR TB patient had lived.

**Figure 2:**
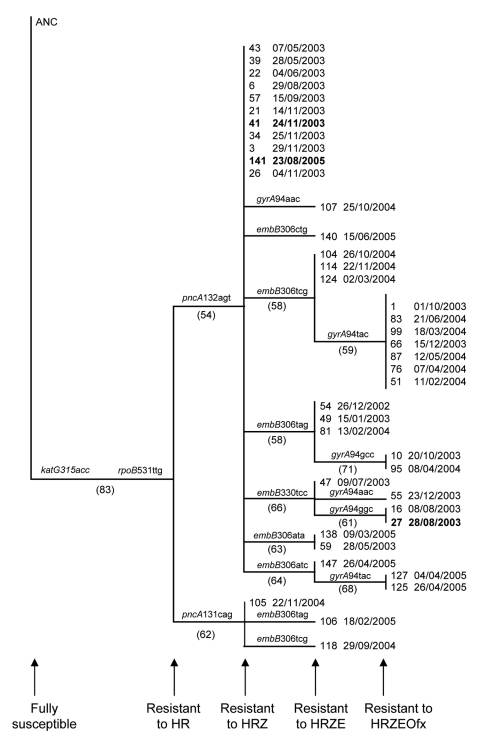
Phylogenetic history of the largest multidrug-resistant tuberculosis (MDR TB) cluster, South Africa, 2003–2005. Genetic data from isolates from 40 of the 42 case-patients were analyzed. The phylogenetic tree was constructed by using the neighbor joining algorithm (PAUP 4.0*; Sinauer Associates, Sunderland, MA, USA) and was rooted to the H37Rv wild-type DNA sequence (ANC) (*20*). The gene and the codon conferring resistance are indicated at the internal node where they occurred. Bootstrap values are shown in brackets at the internal nodes. The sequential evolution of resistance to HRZE and Ofx is indicated. The date of MDR TB diagnosis follows each case number. The 3 XDR TB cases are indicated in **boldface**. H, isoniazid, R, rifampin, E, ethambutol, Z, pyrazinamide, Ofx, ofloxacin.

Phylogenetic reconstructions of the isolates included in the largest identified a single genetically distinct progenitor MDR TB strain (Figure 2). This strain acquired resistance to pyraziamide on 2 separate occasions and both of these strains were subsequently transmitted. Thereafter, ethambutol resistance evolved independently in several different cases. Sequencing of the *gyrA* gene showed that ofloxacin resistance subsequently evolved on 6 separate occasions, resulting in 15 cases of pre–XDR TB. One of these pre–XDR TB strains then evolved to XDR TB and caused disease in a single patient (patient 27). An additional XDR TB strain evolved independently (lacking *gyrA* mutations) (patient 41) and subsequently spread to a contact (patient 141).

## Discussion

Using a combination of clinical, epidemiologic, and molecular data, we showed that drug-resistance was primarily transmitted in this mine setting, thereby suggesting that the current TB control program was largely able to prevent the acquisition of drug-resistance to at least the first-line anti-TB drugs could not prevent the transmission of preexisting MDR TB in this highly vulnerable population. Similar findings have been reported for community based settings in South Africa (*21*,*22*). Our results also demonstrated that a large proportion of patients who had a previous documented episode of TB were reinfected with a circulating MDR TB strain. These findings are consistent with those from another recent study which showed that patients in China with drug-resistant TB who had previously received treatment were frequently infected with a clustered (i.e., recently transmitted) strain (*23*). In previous work, we demonstrated that a prior episode of TB may increase the risk of a subsequent episode of disease through reinfection, even in a population with a low HIV prevalence (*24*). This heightened susceptibility may be especially severe in HIV co-infected patients, given previous concerns that TB may accelerate immune suppression (*25*).

In this study, a large proportion of the HIV-infected TB patients had smear-positive TB, despite the fact that many had CD4 counts <200 cells/mm^3^. These data are consistent with previous findings that suggest that 35% of TB cases in HIV-infected persons are smear negative (*26*). Most notably, this finding demonstrated that in this high risk environment, HIV co-infected patients can transmit TB to close and susceptible contacts, even in the setting of a vigorous TB control program. Of note, we found that active case finding by biannual chest radiographic screening identified only 30% of TB cases

Our phylogenetic reconstruction of the largest cluster of cases demonstrated sequential acquisition of resistance-causing mutations. We believe that the evolution of resistance to ethambutol and pyrazinamide represents the further amplification of drug resistance in the context of patients with undiagnosed MDR TB initially being given standard therapy (*27*). An MDR TB case-patient with a strain resistant to isoniazid, rifampin, ethambutol, and pyrazinamide could then spread disease to persons who were co-hospitalized for drug-susceptible TB or illnesses other than TB. Disease may develop in these persons, and they can then spread MDR TB to their contacts at their place of work or residence, thereby unintentionally perpetuating the drug-resistant TB outbreak. We believe that this observation is not unique to this setting (*12*,*28*–*32*).

Our phylogenetic analysis also indicated that ofloxacin resistance emerged on many occasions after acquisition of resistance to first-line drugs. Although these data suggest that treatment of MDR TB patients with second-line drugs resulted in the evolution of ofloxacin resistance, the mechanisms by which patients acquired this resistance, despite excellent adherence to MDR TB treatment, remain unclear. This stresses the need for pharmacokinetic studies to optimize dosages and treatment regimens for MDR TB in HIV co-infected patients and HIV uninfected patients.

In conclusion, we recommend that DOTS and DOTS-Plus TB control programs (*33*) should be integrated with well functioning HIV management programs to ensure that ARVs are widely administered to limit susceptibility to TB disease. Furthermore, additional intervention measures are required to identify infectious cases. Such measures should include, increasing public awareness of TB symptoms, active screening of all patients making contact with the health care services and more aggressive case finding. The high proportion of smear positive cases with drug-resistant TB suggests that more frequent sputum smear examinations may allow for the early identification these infectious cases. In addition, more frequent culture based diagnosis may identify cases before they become infectious. This study also emphasizes the importance of the development and implementation of rapid DST diagnostics to minimize the delay in detecting MDR TB and the risk of inadvertently placing the patient on a regimen that could lead to the amplification of drug-resistance (*34*). Rapid DST diagnostics may help to prevent nosocomial spread of MDR TB since patients could be rapidly identified and isolated from others (*26*). Our finding that a large proportion of patients in the largest cluster were hospitalized at the same time, raises the possibility that transmission was nosocomial. This may be curtailed by more rigorous infection control measures. We recommend that adequate infection control measures should be implemented in all hospital departments and gathering places to prevent nosocomial infections.

## Supplementary Material

Appendix Figure Genotype and phenotype classification drug-resistant isolates from each case-patient. Insertion sequence (IS) 6110 DNA fingerprints of a single Mycobacterium tuberculosis isolate from 122 case-patients, South Africa, 2003-2005 are shown. Spoligotype patterns from 126 case-patients are shown. Isolated from 74 case-patients were grouped into 11 clusters (4 clusters had 2 cases, 4 clusters had 3 cases, 1 cluster had 4 cases, 1 cluster had 8 cases and 1 cluster had 42 cases). Mycobacterial interspersed repetitive unit (MIRU) types are shown for strains with <5 IS6110 hybridizing bands. Drug-resistant phenotypes of each isolate are illustrated: H, isoniazid, R, rifampin, E, ethambutol, Z, pyrazinamide, Et, ethionamide, S, streptomycin, K, kanamycin, Ofx, ofloxacin. Drug-resistance classification for each case is indicated. RFLP, restriction fragment length polymorphism; NA, not available.
