# Supplementary figures and images for: Emergence of Increased Resistance and Extensively Drug-Resistant Tuberculosis Despite Treatment Adherence, South Africa
Source: Emerg Infect Dis. 2010 Feb;16(2):264–71. doi: 10.3201/eid1602.090968 (PMC2958014; doi:10.3201/eid1602.090968)

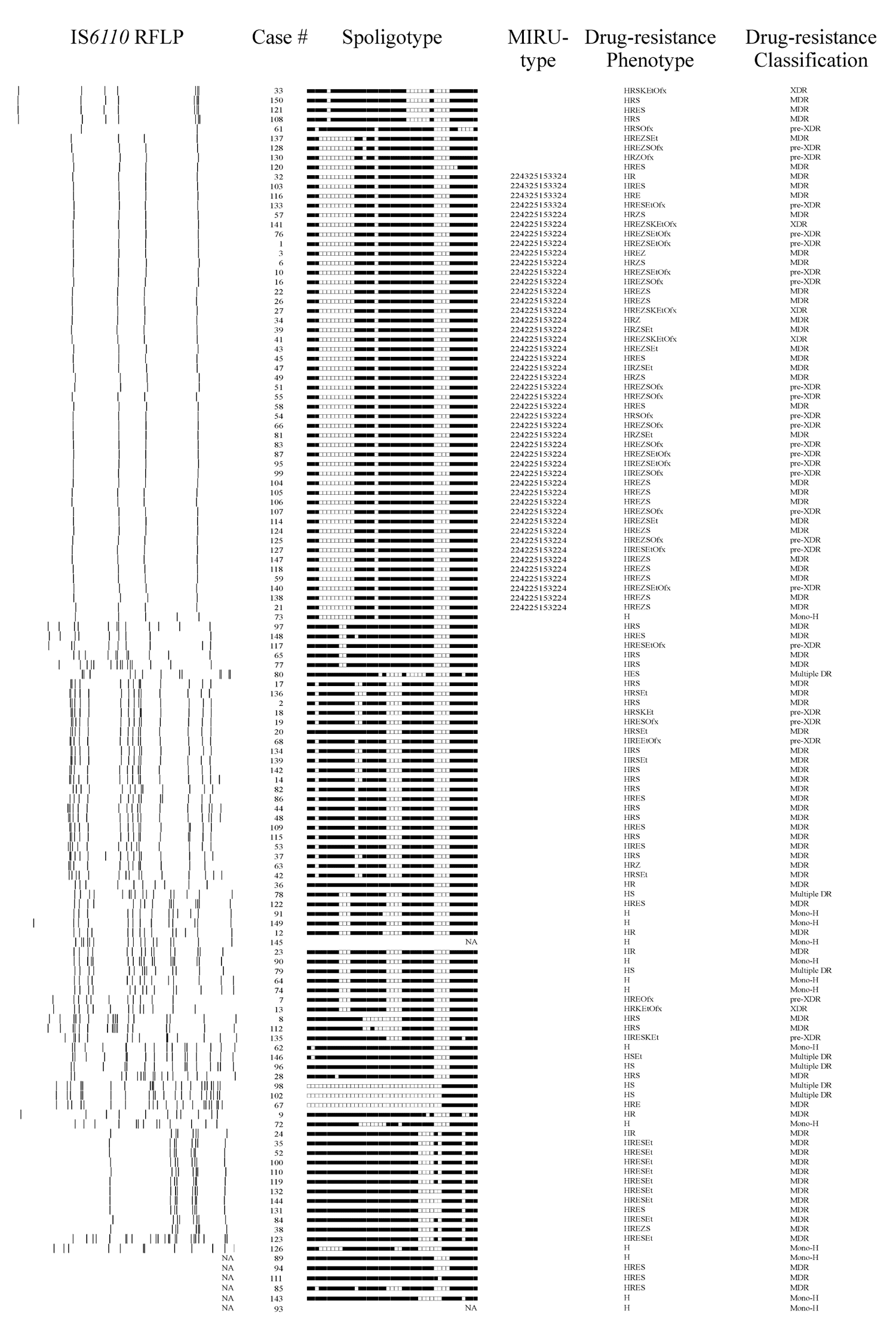

Supplement: Appendix Figure — Genotype and phenotype classification drug-resistant isolates from each case-patient. Insertion sequence (IS) 6110 DNA fingerprints of a single Mycobacterium tuberculosis isolate from 122 case-patients, South Africa, 2003-2005 are shown. Spoligotype patterns from 126 case-patients are shown. Isolated from 74 case-patients were grouped into 11 clusters (4 clusters had 2 cases, 4 clusters had 3 cases, 1 cluster had 4 cases, 1 cluster had 8 cases and 1 cluster had 42 cases). Mycobacterial interspersed repetitive unit (MIRU) types are shown for strains with <5 IS6110 hybridizing bands. Drug-resistant phenotypes of each isolate are illustrated: H, isoniazid, R, rifampin, E, ethambutol, Z, pyrazinamide, Et, ethionamide, S, streptomycin, K, kanamycin, Ofx, ofloxacin. Drug-resistance classification for each case is indicated. RFLP, restriction fragment length polymorphism; NA, not available. [file 09-0968_appF-s1.gif]
